# Contrastive Learning with Prototype-Based Negative Mixing for Satellite Telemetry Anomaly Detection

**DOI:** 10.3390/s23104723

**Published:** 2023-05-13

**Authors:** Guohang Guo, Tai Hu, Taichun Zhou, Hu Li, Yurong Liu

**Affiliations:** 1 National Space Science Center, Chinese Academy of Sciences, Beijing 101499, China; 2 Department of Computer Science and Technology, University of Chinese Academy of Sciences, Beijing 101408, China

**Keywords:** telemetry data, anomaly detection, contrastive learning, negative mixing

## Abstract

Telemetry data are the most important basis for ground operators to assess the status of satellites in orbit, and telemetry data-based anomaly detection has become a key tool to improve the reliability and safety of spacecrafts. Recent research on anomaly detection focuses on constructing a normal profile of telemetry data using deep learning methods. However, these methods cannot effectively capture the complex correlations between the various dimensions of telemetry data, and thus cannot accurately model the normal profile of telemetry data, resulting in poor anomaly detection performance. This paper presents CLPNM-AD, contrastive learning with prototype-based negative mixing for correlation anomaly detection. The CLPNM-AD framework first employs an augmentation process with random feature corruption to generate augmented samples. Following that, a consistency strategy is employed to capture the prototype of samples, and then prototype-based negative mixing contrastive learning is used to build a normal profile. Finally, a prototype-based anomaly score function is proposed for anomaly decision-making. Experimental results on public datasets and datasets from the actual scientific satellite mission show that CLPNM-AD outperforms the baseline methods, achieves up to 11.5% improvement based on the standard $F_{1}$ score and is more robust against noise.

## 1. Introduction

Satellites are currently one of the most sophisticated technological systems, performing their mission in harsh conditions [1]. It is unlikely to be able to entirely dismiss the possibility of faults due to factors such as inadequate design verification, extreme space environment, damage accumulation effect and dynamic change of on-board state [2,3], and serious anomalies will lead to mission degradation or even failure. Telemetry data reflect the health condition of the corresponding device and are the most important basis for ground operators to determine the status of the satellite in orbit [4]. As a result, techniques for mining telemetry data and detecting anomalies have been developed to lessen the monitoring burden on ground operators while also improving the reliability and safety of satellites in orbit.

Anomaly detection is a technique for identifying unexpected patterns of deviation from normal behavior and has attracted a significant amount of research attention in recent years [5,6,7]. Satellites are usually monitored by numerous sensors, each of which measures a distinct variable. Monitoring the devices or subsystems with correlations between multiple monitoring indicators is critical to ensuring the normal operation of the satellite [8,9]. In satellite anomaly detection settings, anomaly data are relatively rare and other new types of anomalies are continually being discovered over time, owing to changing environmental factors and command sequences, so a supervised approach is not applicable in this case. In contrast, normal data are easily obtained, so we concentrate on identifying correlation anomalies using a semi-supervised approach, which means that all of the training data are normal data.

Semi-supervised anomaly detection methods use a significant amount of normal data to create a normal profile of correlation between variables. Samples with behaviors deviate from the normal pattern are labeled as anomalies. Recently, deep anomaly detection methods have received a significant amount of attention, as they primarily employ well-designed encoders to capture complex correlations between variables, such as the autoencoder-based method [10,11,12,13], generative adversarial networks-based method [14,15,16], self-supervised anomaly detection method [17,18], and so on. The reconstruction error or sample features obtained from the learned encoder are then used to detect anomalies. However, due to the complex and frequently dynamically changing patterns of correlations between the variables of the telemetry data, the encoder maps normal and abnormal samples into the feature space, making them overlapping and difficult to distinguish, failing to accurately construct normal profiles of normal data, and resulting in unsatisfactory abnormality detection results.

In this paper, we propose an anomaly detection method called contrastive learning with prototype-based negative mixing for anomaly detection (CLPNM-AD). Contrastive learning aims to learn a transformation-invariant feature representation and evenly distributes the sample on the hypersphere in representation space [19,20]. Combining encoders with contrastive loss to model the correlations of normal data, we find that the learned representation can distinguish between normal and abnormal patterns. However, with hardness-aware property [21], the contrastive loss imposes larger gradient magnitudes on samples closer to the anchor, leading the semantically similar samples (false negative samples) to separate, disrupting the local semantic structure between samples. Therefore, we devise a prototype-based hard negative mixing strategy to guide network learning to ensure that the local semantic structure between samples is not disrupted, thereby constructing a more distinguishing profile for normal data.

Going beyond previous anomaly detection methods, CLPNM-AD can capture complex correlations between telemetry data variables, maintaining a local semantic structure while pushing samples with different semantics far apart, making normal and anomaly samples as separable as possible. Specifically, we first employ an augmentation process with random feature corruption to obtain augmented samples. Following that, a prototype consistency strategy is used to capture the semantic category information of the samples. After that, a prototype-based negative mixing contrast loss is used to obtain a normal profile. Unlike the vanilla contrast loss, which maps all samples evenly onto the hypersphere, the proposed method will make the true negative samples from all directions around the anchor point (excluding the false negative samples) have an effective gradient such that these samples are separable from the anchor, making normal and anomaly samples distinguishable. Finally, anomaly decisions are made using a prototype-based anomaly score function. Extensive experiments are conducted to verify the effectiveness of the proposed method.

The contributions of this paper are summarized below:

- We propose CLPNM-AD, a correlation anomaly detection method, which combines prototype consistency and prototype-based contrastive loss to guide the encoder to construct an accurate normal profile, making normal and anomaly samples more distinguishable and thus facilitating the detection of anomalies.
- We apply a sample augmentation process with random feature corruption to generate positive samples for contrast learning and to help capture the complex correlations between variables.
- We combine prototype consistency and prototype-based contrast loss to learn features that facilitate the distinguishing of normal and anomalous samples. First, prototype consistency is proposed to capture the semantic categories of samples. Then, a prototype-based hard negative mixing strategy is applied to preserve local semantic information and push samples of different prototypes farther apart.
- We propose an anomaly score function that indicates the degree of the anomaly of a sample by calculating the Mahalanobis distance between the sample and the prototype conditional Gaussian distribution.
- We conduct extensive experiments to evaluate the performance of CLPNM-AD on three public datasets and one satellite telemetry dataset from our actual mission. The experiment results show the excellent performance of the proposed method.

The remainder of the paper is organized as follows: Section 2 describes related work. Section 3 introduces the anomaly categories as well as the fundamental concepts of contrastive learning. Section 4 describes the proposed method CLPNM-AD in detail. Section 5 describes the experiment findings and analysis. Section 6 provides a summary of the paper.

## 2. Related Work

### 2.1. Anomaly Detection

Anomaly detection of satellite telemetry data has recently become a popular research topic, with a large body of research literature emerging. Traditional anomaly detection methods have mainly used statistical or machine learning methods to detect anomalies. In [22], a kernel density estimates-based model was proposed, and samples with low density were labeled as anomalies. In [23], the support vector machine was used to build a hyperplane to keep normal data away from the origin where samples close to the origin were detected as anomalies. In [24], Lishuai Li et al. proposed a clustering method based on a Gaussian mixture model to detect unusual data patterns in flight data, where samples with low probability were detected as anomalous. In [25], the authors labeled samples from low-density regions as anomalous. Although these traditional anomaly detection methods are efficient in most cases, they do not work well when the data dimension is too high and the relationships between variables are too complex.

Self-supervised representation learning methods use the data themselves to construct supervised information to achieve data representation learning. In recent years, self-supervised deep anomaly detection methods have made significant progress, improving anomaly detection performance and gaining much attention. In [26], an encoder was used to map the normal samples into a hypersphere to model the representation of the normal pattern. In [11], the authors used an auto-encoder to learn the features of the samples, which were subsequently combined with reconstruction errors to be fed into a Gaussian mixture model to generate anomaly scores. In [17], the data samples were first augmented with random affine transformation, followed by a classification model for anomaly detection. In [27], the authors used contrastive loss to obtain an encoder and then fed the features generated by the encoder into a one-class classifier for anomaly detection. In [28], the authors first created distributionally shifted augmentations, then trained the encoder with contrastive loss to obtain sample features, and finally detected anomalies based on the cosine distance between the learned features. In [18], trainable augmentation processes were used to obtain augmented samples, and then an end-to-end anomaly detection pipeline was built by contrastive loss.

### 2.2. Contrastive Learning

Contrastive learning as a type of self-supervised method aims to learn a transformation-invariant feature representation such that multiple views (also known as augmentations) of a sample keep attracting while repelling to other samples [20]. In [29], the authors proposed a feature learning method using contrastive predictive coding, which uses an autoregressive model to predict future values in the hidden space, and an InfoNCE loss function for model training. Ref. [30] proposed an unsupervised feature learning method that constructed an instance discrimination task via a memory bank and learned sample features using a noise contrastive estimation loss function. In [31], the authors further considered the negative samples selection strategy and constructed a contrast task by obtaining negative samples from a dynamic dictionary instead of a memory bank. In [19], the authors discussed the role of sample augmentation and added a non-linear layer in the middle of the representations and contrastive loss to improve the quality of the learned features. In [32], the authors used prototype vectors as the subject of contrasting rather than the instances themselves, learned features through classification consistency between views, and explored augmentation methods to improve the quality of the learned representation.

### 2.3. Debiased Negatives Sampling

Contrastive learning learns features by comparing similar or dissimilar pairs of samples, so the quality of positive and negative samples determines the quality of the learned features. Contrastive learning is a self-supervised representation learning method that does not have access to sample labels. When anchor samples and negative samples form negative pairs, these samples may belong to the same semantic class, and the hardness-aware property of contrastive learning will cause these samples to be repelled, destroying the local semantic structure and affecting representation learning. These false negative samples continue to be a major issue in contrastive learning, but relatively little research work has been conducted in this area.

In [33], the authors proposed a method for synthesizing hard negative samples in the feature space by selecting the K closest negative samples to the anchor and randomly mixing them. Ref. [34] established the distribution of difficult negative samples and proposed an importance sampling strategy, based on which the authors designed a new contrastive loss with adjustable hyperparameters to allow users to control the hardness. To lessen the impact of false negatives, ref. [35] offered a debiased contrastive loss, and [36] proposed a negative elimination and false attraction loss. In [37], the authors proposed a modified contrastive loss that eliminates false negative samples and removes negative samples that are similar to the anchors. To address the sampling bias problem, ref. [38] obtained the semantic structure of graph data by clustering and then weighted the negative samples according to the distance between semantic categories.

## 3. Preliminaries

### 3.1. Categories of Anomalies in Telemetry Data

**Figure 1:**
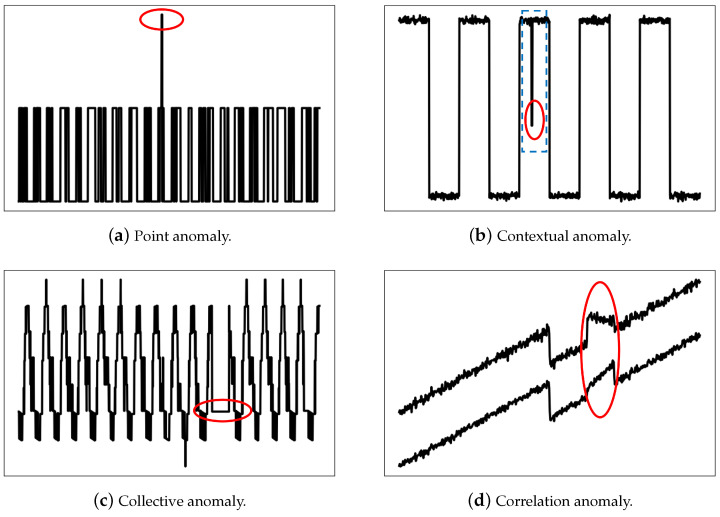
Examples of anomalies in satellite telemetry data (anomalies highlighted in red circles and time window in the blue box).

Anomalies in satellite telemetry data are classified as point anomalies, contextual anomalies, collective anomalies and correlation anomalies, as illustrated schematically in Figure 1.

Point anomalies refer to a single data point or several consecutive data points that deviate significantly from other data points. Figure 1a depicts an example of a point anomaly in which the point in the red circle extends beyond the other points.

Contextual anomalies mean that the data points deviate a local threshold determined by the temporal context, although not deviating the global threshold. Figure 1b shows an example of a contextual anomaly where the point in the red circle deviates from the other samples in the time window identified by the blue box.

Collective anomalies are consecutive points that do not exceed a threshold, but the subsequence formed by these points violates the pattern of the original telemetry time series. As shown in Figure 1c, the sequence of consecutive points in the red circle is a collective anomaly, which has a different shape to the rest of the sequence.

Correlation anomalies occur when the stable correlation between multiple telemetry parameters is broken and the correlation between parameters exhibit different patterns. Correlations between satellite telemetry parameters include physical correlations, logical correlations and operational mode correlations. As shown in Figure 1d, there is a positive correlation between the two parameters in most cases, but in the red circle, the correlation becomes negative, indicating a correlation anomaly.

The correlations between satellite telemetry parameters in real-world scenarios can be very complex, necessitating anomaly detection methods capable of modeling such complex correlations. The objective of this paper is primarily to propose a correlation anomaly detection method that is sensitive to abnormal changes in the correlation between satellite telemetry parameters.

### 3.2. Contrastive Learning

Contrastive learning learns representations by grouping differently augmented views of the same data sample together [19]. During the training process, a mini-batch with size *N* is randomly selected, and the contrastive loss is defined on augmented views generated from the mini-batch. Let $x_{i}^{\left(1\right)}=A^{\left(1\right)}\left(x_{i}\right)$ and $x_{i}^{\left(2\right)}=A^{\left(2\right)}\left(x_{i}\right)$, where $A^{\left(1\right)}$ and $A^{\left(2\right)}$ are independent stochastic augmentation functions. Following [19,27], we define contrastive loss as follows:

(1) $$\begin{array}{cc} L_{infonce} & (x_{i}^{\left(1\right)},x_{i}^{\left(2\right)})=\\ \; & -\frac{1}{N}\sum_{i=1}^{N}\log\frac{exp(dist\left(z_{i}^{\left(1\right)},z_{i}^{\left(2\right)}\right)/\nu )}{\sum_{j=1}^{N}1_{\left[j\neq i\right]}exp\left(dist\left(z_{i}^{\left(1\right)},z_{j}^{\left(1\right)}\right)/\nu \right)+\sum_{j=1}^{N}exp\left(dist\left(z_{i}^{\left(1\right)},z_{j}^{\left(2\right)}\right)/\nu \right)}, \end{array}$$

where $dist\left(u,v\right)=\frac{u^{T}v}{\left||u||||v|\right|}$, $z_{i}^{\left(1\right)}=h\circ f\left(x_{i}^{\left(1\right)}\right)$, $z_{i}^{\left(2\right)}=h\circ f\left(x_{i}^{\left(2\right)}\right)$, f(·) is an encoder, h(·) is a neural network layer that transforms feature vectors to the space where contrastive loss is used, $1_{\left[j\neq i\right]}$ represents an indicator function that outputs 1 when j≠i holds and 0 otherwise and ν is a temperature parameter.

## 4. Proposed Method

We first present the problem definition of satellite telemetry data correlation anomaly detection. Then, we give an overview of CLPNM-AD and briefly introduce its modules. Finally, we describe the proposed method in detail.

### 4.1. Problem Definition

This paper is based on the assumption that, in routine operation, correlations between satellite telemetry variables exhibit some common patterns, and that ground operators should focus on the few outliers that deviate from these common patterns.

Each sample of satellite telemetry data from a subsystem or device at time *t* is represented by a vector, as in the form

(2) $$x_{t}=\left[d_{t}^{1},d_{t}^{2},\ldots ,d_{t}^{M}\right],$$

where $d_{t}^{j}$ is the value of the *j*-th dimension of telemetry data at time *t* and *M* is the dimension of sample $x_{t}$.

The semi-supervised scenario is addressed in this paper, in which the training dataset $X_{train}=\left\{x_{1},x_{2},\ldots ,x_{N}\right\}$ contains only normal data samples and the test dataset $X_{test}$ contains both normal and abnormal data samples. The goal of the anomaly detection method is first to create an anomaly scoring model s(·) from $X_{train}$:

(3) s:x→b,

where *b* is the anomaly score that predicts how anomalous the sample *x* is, and then to create an anomaly decision function Δ(·) to emit a judgment of whether *x* is normal or abnormal:

(4) Δ:b→{0,1},

where 0 denotes normal, 1 denotes anomalous and the anomaly decision function Δ(·) is often implemented by a conditional judgment, which means that the sample *x* is determined to be anomalous if its anomaly score, s(x), exceeds a specified threshold.

### 4.2. Overview of CLPNM-AD Framework

**Figure 2:**
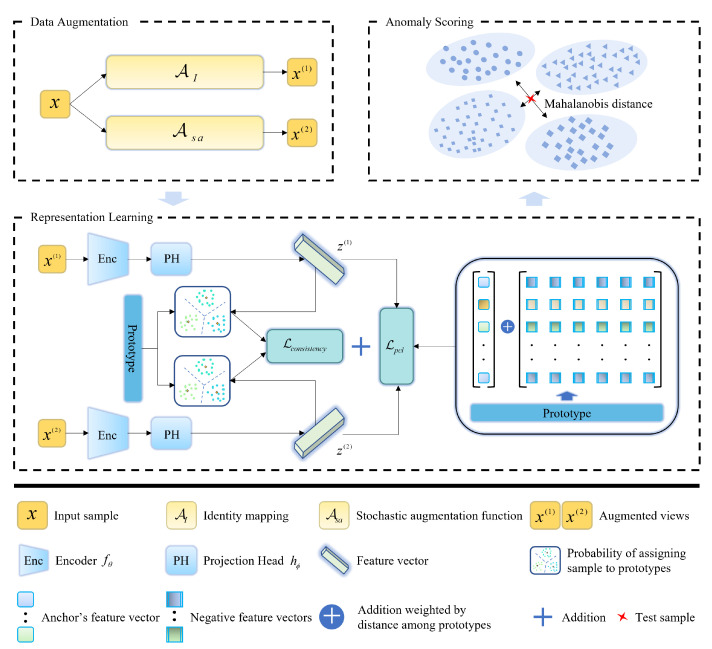
The framework of CLPNM-AD.

The CLPNM-AD framework is shown in Figure 2, which contains a data augmentation module, representation learning module and anomaly scoring module.

First, we use the augmentation functions $A_{I}$ and $A_{sa}$ to generate two augmented samples of sample *x*. We then propose a contrastive learning with the prototype-based negative mixing (CLPNM) method to learn the representation of the training data $X_{train}$. As depicted in the representation learning module in Figure 2, the encoder and projection head map the augmented samples to the feature space, then the probability values of the feature vectors assigned to the prototypes are obtained to learn the semantic consistency of the augmented samples and finally the hard negative samples are generated to guide the contrastive representation learning. In this way, a representation specific to anomaly detection is obtained. Finally, the anomaly score is calculated for each sample based on the Mahalanobis distance between the sample feature and the closest prototype.

### 4.3. Data Augmentation

As a vital part of contrastive learning, techniques to generate views are domain-specific (e.g., color distortion [19] and geometric transformation [32] in computer vision). Unlike image data, telemetry data cannot be augmented using color and geometric transformations. In this paper, two augmentation strategies, $A_{I}$ and $A_{sa}$, are used as augmentation functions, where $A_{I}$ is a identity mapping and $A_{sa}$ is a stochastic process with random feature corruption.

Inspired by the literature [39], the stochastic augmentation generation method is shown in Algorithm 1. In this algorithm, we denote a process $A_{sa}$ to obtain the augmentation view of a sample *x*. It first computes the empirical marginal distribution for each feature’s values throughout the whole training dataset, and next randomly selects a subset of features and draws a random value from the empirical marginal distribution of each feature in that subset to replace the value of that feature in the sample.
**Algorithm 1:** Stochastic augmentation function $A_{sa}$
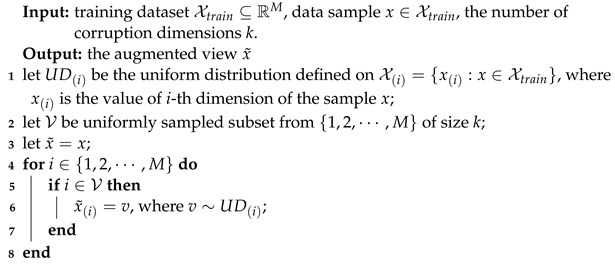


### 4.4. Representation Learning

In the CLPNM-AD method, a one-dimensional convolution encoder maps the samples into the feature space. The prototype and pseudo-label of the sample are then calculated by self-labeling based on the features. Finally, hard negatives are synthesized based on the prototypes and pseudo-labels to guide the network parameters learning. Prototype learning and network parameters updating are performed alternately.

#### 4.4.1. One-Dimensional Convolution Encoder

**Figure 3:**
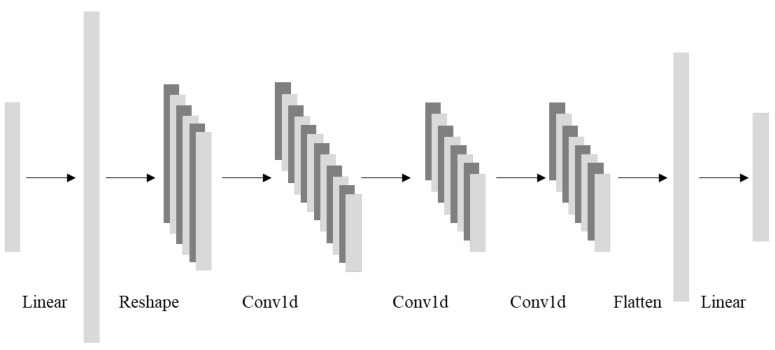
One-dimensional convolutional network structure.

The main goal of the encoder is to map the samples to the feature space. As an encoder in this paper, a one-dimensional convolutional network is designed, as shown in Figure 3. To begin, a linear layer is used to map the samples to a high-dimensional vector, which is then reshaped to obtain the features of multiple channels, after which one-dimensional convolutional layers are used to perform convolutional operations, and finally the features of multiple channels are flattened to obtain the final feature vector of the samples.

#### 4.4.2. Self-Labeling by Clustering Consistency

As shown in Figure 2, deep neural networks h∘f(·) map the augmented view $x_{i}^{\left(a\right)}$ to the feature vector $z_{i}^{\left(a\right)}\in R^{D}$ by $z_{i}^{\left(a\right)}=h\circ f\left(x_{i}^{\left(a\right)}\right)$, where a∈{1,2} is the index of a view. After that, the feature vector $z_{i}^{\left(a\right)}$ is mapped to *K* clusters, the centroids of which are implemented by *K* trainable prototype vectors $\left\{c_{1},c_{2},\ldots ,c_{K}\right\}$. We define matrix *C*, whose columns are composed of the vectors $c_{1},c_{2},\ldots ,c_{K}$, and which is implemented by a single linear neural network. The matrix *C* converts the feature vector $z_{i}^{\left(a\right)}$ to class scores. The softmax operator is then used to map the class scores to the class probabilities:

(5) $$p\left(y=\cdot |x_{i}^{\left(a\right)}\right)=softmax\left(C\circ h\circ f\left(x_{i}^{\left(a\right)}\right)\right).$$

We represent the pseudo-label of the sample $x_{i}^{\left(a\right)}$ by posterior distribution $q(y=\cdot |x_{i}^{\left(a\right)})$. The cross-entropy loss of $p(y=\cdot |x_{i}^{\left(a\right)})$ and $q(y=\cdot |x_{i}^{\left(a\right)})$ defines as

(6) $$E\left(p\left(x^{\left(a\right)}\right),q\left(x^{\left(a\right)}\right)\right)=-\frac{1}{N}\sum_{i=1}^{N}\sum_{y=1}^{K}q\left(y|x_{i}^{\left(a\right)}\right)\log p\left(y|x_{i}^{\left(a\right)}\right).$$

In the anomaly detection setting, the semantic label of the sample is unknown. Optimizing $E(p\left(x^{\left(a\right)}\right),q\left(x^{\left(a\right)}\right))$ will lead *q* to a degenerate solution, namely assign all samples to a randomly single pseudo-label. To prevent degenerate solutions, we introduce a constraint that the assignment of pseudo-labels must divide the data samples from a mini-batch into clusters with equal size [40]. Formally, the optimization objective is thus

(7) $$\min_{p,q}E\left(p\left(x^{\left(a\right)}\right),q\left(x^{\left(a\right)}\right)\right)\;s.t.\;\forall y:q\left(y|x_{i}^{\left(a\right)}\right)\;\in \left[0,1\right]\;and\;\;\sum_{i=1}^{N}q\left(y|x_{i}^{\left(a\right)}\right)=\frac{N}{K}.$$

The objective of Equation (7) can be viewed as an *optimal transport problem*. To solve this problem, we define *P* and *Q* as two K×N matrices of joint probability. The elements in *P* and *Q* denote as

(8) $$P_{yi}=p\left(y|x_{i}^{\left(a\right)}\right)\frac{1}{N};\;Q_{yi}=q\left(y|x_{i}^{\left(a\right)}\right)\frac{1}{N}.$$

To meet the equal partition constraint, we make the matrix *Q* a *transportation polytope* [40]:

(9) $$U=\{Q\in R_{+}^{K\times N}\;|\;Q1_{N}=\frac{1}{K}1_{K},\;Q^{T}1_{K}=\frac{1}{N}1_{N}\},$$

where $1_{N}$ and $1_{K}$ are vectors of all ones of corresponding dimensions.

Then, the optimization objective in Equation (7) can thus be expressed as

(10) $$\min_{Q\in U}\langle Q,-\log P\rangle ,$$

where 〈·〉 denotes the Frobenius dot-product of two matrices. The Sinkhorn–Knopp [41] algorithm is then used to solve the above transport problem, which amounts to introducing a regularization term

(11) $$\min_{Q\in U}\langle Q,-\log P\rangle +\frac{1}{N}KL(Q||rc^{T}),$$

where KL denotes the Kullback–Leibler divergence, $r=\frac{1}{K}1_{K}$, and $c=\frac{1}{N}1_{N}$. The advantage of bringing in the regularization term $KL(Q||rc^{T})$ is that the minimizer of Equation (11) can be transformed to

(12) $$Q=diag\left(\Gamma \right)P^{\lambda }diag\left(\Lambda \right),$$

where Γ and Λ are two scaling coefficient vectors and λ is used to balance the convergence speed and proximity level to the original transport problem. In this paper, we specify a fixed value for λ.

The pseudo-label matrix *Q* is then used to guide the learning of the neural network parameters. To encourage the two augmented views $x_{i}^{\left(1\right)}$ and $x_{i}^{\left(2\right)}$ to be assigned to the same cluster, we assign $x_{i}^{\left(1\right)}$ the pseudo-label $q(y=\cdot |x_{i}^{\left(2\right)})$ (not $q(y=\cdot |x_{i}^{\left(1\right)})$) and vice versa. The consistency loss denotes as

(13) $$L_{consistency}=-\frac{1}{N}\sum_{i=1}^{N}\sum_{y=1}^{K}\;\left[\;q\left(y|x_{i}^{\left(1\right)}\right)\log p\left(y|x_{i}^{\left(2\right)}\right)+q\left(y|x_{i}^{\left(2\right)}\right)\log p\left(y|x_{i}^{\left(1\right)}\right)\;\right],$$

where *N* denotes the mini-batch size and *K* denotes the number of prototypes.

#### 4.4.3. Negative Mixing Contrastive Objective

The pseudo-label matrix *Q*, on one hand, is used to learn clustering consistency, and on the other hand, is used to mitigate the sampling bias and obtain hard negatives to guide the contrastive loss. The soft label is represented by *Q* in the preceding section, while we require the hard label in this part to reflect the semantic clusters of the data. To acquire the hard labels, we simply apply the argmax function to the soft labels.

We obtain hard negatives by mixing the anchor feature vector with sample vectors from different prototypes in the mini-batch. The weight of sample mixing is determined by the distance between prototypes. We denote the distance between two prototypes as

(14) $$d\left(c_{i},c_{j}\right)=1-\frac{c_{i}^{T}c_{j}}{\left|\right|c_{i}{\left|\right|}_{2}\left|\right|c_{j}{\left|\right|}_{2}},$$

where $c_{i}$ and $c_{j}$ are two prototypes from prototypes *C* in Figure 2.

For the anchor feature vector $z_{i}$ and sample feature vector $z_{j}$, the mixed sample can be calculated by

(15) $$h_{j}=\frac{{\tilde{h}}_{j}}{\left|\right|{\tilde{h}}_{j}{\left|\right|}_{2}};{\tilde{h}}_{j}=z_{i}\cdot \left(1-d\left(c_{i},c_{j}\right)\right)+z_{j}\cdot d\left(c_{i},c_{j}\right).$$

The prototype-based contrastive loss is defined as

(16) $$L_{pcl}=-\frac{1}{N}\sum_{i=1}^{N}\log\left\{\frac{exp(dist\left(z_{i}^{\left(1\right)},z_{i}^{\left(2\right)}\right)/\nu )}{\sum_{j=1}^{2N}1_{c_{i}\neq c_{j}}exp\left(dist\left(z_{i}^{\left(1\right)},h_{j}\right)/\nu \right)}+\frac{exp(dist\left(z_{i}^{\left(2\right)},z_{i}^{\left(1\right)}\right)/\nu )}{\sum_{j=1}^{2N}1_{c_{i}\neq c_{j}}exp\left(dist\left(z_{i}^{\left(2\right)},h_{j}\right)/\nu \right)}\right\}.$$

The final training objective couples $L_{consistency}$ and $L_{pcl}$ as

(17) $$L=L_{pcl}+\eta \;L_{consistency},$$

where η is used to balance $L_{pcl}$ and $L_{consistency}$.

The core of the proposed method is to learn a model C∘g∘f and a pseudo-label matrix *Q*. This is accomplished by alternately performing the following two steps:

**Step 1: representation learning.** Given the pseudo-label assignments matrix *Q*, the parameters of model C∘g∘f are optimized by minimizing Equation (17).

**Step 2: self-labeling.** Given the model C∘g∘f obtained from step 1, we first calculate the class probability *P* by using Equation (5), and then calculate *Q* using Equation (12):

(18) $$\forall y:\Gamma_{y}={\left[P^{\lambda }\Lambda \right]}_{y}^{-1};\;\forall i:\Lambda_{i}={\left[\Gamma^{T}P^{\lambda }\right]}_{i}^{-1}.$$

### 4.5. Score Functions for Detecting Anomaly

To determine whether a sample is normal or anomalous, we use representations and prototypes to assess the degree of anomaly of the sample. Refs. [27,42] show that the projection head focuses too much on the proxy task, and the output features lose useful information for the downstream task. Therefore, we construct the anomaly score function based on the representations of encoder $f_{\theta }$.

Let $x_{i}$ be an input and $y_{i}\in \left\{1,\cdots ,K\right\}$ be its label, which is obtained by executing argmax operation on the pseudo-label matrix *Q* in Section 4.4.2. Following [43,44], we assume that the feature vectors with the same prototype follow a prototype-conditional multivariate Gaussian distribution. Specifically, we define a conditional Gaussian distribution for each of the *K* prototypes, and all *K* conditional Gaussian distributions share a common covariance Σ: $P\left(f\left(x_{i}\right)|y_{i}=c\right)=N\left(f\left(x_{i}\right)|\mu_{c},\Sigma \right)$, where $\mu_{c}$ is the mean of feature vectors calculated by f(·) with the prototype c∈{1,⋯,K}. To estimate the parameters $\mu_{c}$ and Σ in the conditional Gaussian distribution, we use *N* data samples from the entire training set for the calculation (not the samples in a batch):

(19) $${\tilde{\mu }}_{c}=\frac{1}{N_{c}}\sum_{i=1}^{N}1_{\left[y_{i}=c\right]}f\left(x_{i}\right);\;\tilde{\Sigma }=\frac{1}{N}\sum_{c=1}^{K}\sum_{i=1}^{N}1_{\left[y_{i}=c\right]}\left(f\left(x_{i}\right)-{\tilde{\mu }}_{c}\right){\left(f\left(x_{i}\right)-{\tilde{\mu }}_{c}\right)}^{T},$$

where *N* denotes the number of samples contained in the training set, $N_{c}$ denotes the number of samples with prototype *c* and $1_{\left[y_{i}=c\right]}$ outputs 1 when $y_{i}=c$ holds, otherwise 0.

Based on the empirical class mean and the covariance of prototype conditional Gaussian distribution obtained above, we define the anomaly score s(x) for sample *x* by the following equation:

(20) $$s\left(x\right)=\min_{c}{\left(f\left(x\right)-{\tilde{\mu }}_{c}\right)}^{T}{\tilde{\Sigma }}^{-1}\left(f\left(x\right)-{\tilde{\mu }}_{c}\right).$$

The anomaly score indicates the degree of abnormality of each sample. The higher the anomaly score, the more abnormal the sample is. To achieve an anomaly decision for the samples, the protocol in the literature [11,17] is used, in which the decision threshold is based on the percentage of anomalies in the test set, e.g., if the percentage of anomalies in the test set is ρ, the ρ of samples with the highest anomaly scores are considered to be anomalous.

## 5. Experiments

In this section, we describe the selected datasets and the baseline methods, and perform a series of experiments to validate the proposed method. Firstly, the proposed method is compared with the state-of-the-art methods and validated by statistical hypothesis testing. Then, the robustness of the method is verified on data with different contamination ratios. Furthermore, the effectiveness of each module of the method is verified by ablation experiments. Finally, sensitivity analyses are also implemented to explore the response of the model to different hyperparameters. The code is available at https://github.com/guoguohang/CLPNM_AD, (accessed on 15 March 2023).

### 5.1. Datasets

We adopt three public datasets: Thyroid, Satellite, Satimage and one actual telemetry data source from the Quantum Science Experiment Satellite, also known as Micius. The statistical information of the datasets is shown in Table 1.

- Thyroid. The dataset is a thyroid disease classification dataset from the outlier detection datasets (OODS) repository (http://odds.cs.stonybrook.edu/thyroid-disease-dataset/, (accessed on 29 March 2023)), which contains six continuous attributes. The original dataset contains three classes. As hyperfunction is a minority, researchers in the field of anomaly detection treat hyperfunction as anomaly class.
- Micius. The dataset is from the telemetry data of China’s Quantum Science Experiment Satellite, also known as Micius (https://doi.org/10.57760/sciencedb.o00009.00042, (accessed on 29 March 2023)), which contains 19 attributes, and the time span is from January 2017 to February 2019. Micius has four operation patterns. As pattern 4 is rare, we treat pattern 4 as anomaly class and the rest as normal class.
- Satellite. The Satellite dataset is integrated by Landsat Satellite from the OODS repository (http://odds.cs.stonybrook.edu/satellite-dataset/, (accessed on 29 March 2023)). Data from three minority categories, 2, 4 and 5, are combined to form the anomaly class, while the remaining classes are combined to form the normal class.
- Satimage. The Satimage-2 dataset is from the OODS repository (http://odds.cs.stonybrook.edu/satimage-2-dataset/, (accessed on 29 March 2023)) and is also integrated from Landsat Satellite. Combining the training and test data in the Landsat Satellite dataset, there are 71 outliers in class 2 and all other classes are merged into a normal class.

### 5.2. Baseline Methods

The following six methods are selected to compare with CLPNM-AD:

- OC-SVM [23]. One-Class Support Vector Machine (OC-SVM) is a classical kernel-based anomaly detection method that uses normal data to learn a decision boundary in order to distinguish normal from anomalous data.
- LOF [25]. Local Outlier Factor (LOF) uses the degree of isolation of a sample relative to its surrounding neighbors as the anomaly score to achieve the distinction between normal and abnormal data samples.
- DAGMM [11]. Deep Autoencoding Gaussian Mixture Model (DAGMM) consists of a compression network and an estimation network. The autoencoder acts as a compression network to map the samples into the feature space. The obtained feature vectors and the reconstruction error of the compression network are fed to the estimation network to obtain the energy score/anomaly score.
- Deep SVDD [26]. Deep Support Vector Data Description (Deep SVDD) is the deep variant of SVDD, which aims to leverage the feature extraction capabilities of deep learning to learn a hypersphere only using normal data.
- GOAD [17]. GOAD is a classification-based method for detecting anomalies for general data, which obtains anomaly scores by training a classifier on a set of random auxiliary tasks.
- NeuTraL AD [18]. Neural Transformation Learning for Deep Anomaly Detection (NeuTraL AD) uses a set of learnable transform to replace the fixed random affine transform in the previous literature and provides an end-to-end anomaly detection method with loss function in a form similar to the contrastive loss function.

### 5.3. Evaluation Metrics

Following the settings in [11,17], the models are trained using a randomly selected subset of 50% of the normal data and are evaluated on the remaining normal data as well as all the anomaly data. We use the mean and standard deviation (σ) of $F_{1}$ score after 20 random splits to compare the anomaly detection performance. The threshold for identifying anomalous samples is determined by the anomaly ratio ρ in Table 1, which indicates that samples with an anomaly score higher than 100*ρ quantiles will be identified as anomaly. We consider the anomaly class to be positive class and define the $F_{1}$ score accordingly. $F_{1}$ score is defined as follows: $F_{1}=\frac{2*Precision*Recall}{Precision+Recall}$, where Precision=$\frac{\left|G\cap R\right|}{\left|R\right|}$, Recall=$\frac{\left|G\cap R\right|}{\left|G\right|}$, *G* denotes the set of real anomalous samples and *R* denotes the set of anomalous samples identified by our method.

### 5.4. Model Configuration

This section describes the network structure as well as the details of model training. In our experiments, all the CLPNM-AD instances are implemented based on the Pytorch framework. For each dataset, the network structure of the encoder, projection head and prototype in CLPNM-AD are shown in Table 2. For the other hyperparameters, the corruption dimension *k* in the stochastic augmentation process $A_{sa}$ is set to 4, 16, 34 and 32 for the datasets Thyroid, Micius, Satellite and Satimage, respectively, the temperature ν in Equation (16) is set to 0.07 and the balance coefficient η in Equation (17) is set to 0.4 for each dataset. All model instances are trained by using mini-batch SGD with a momentum of 0.9. The initial learning rate $LR_{0}$ is set as 0.001 and updated by $LR_{t}=LR_{t-1}*0.5*\left(1+cosine\left(\pi +t/N_{D}\right)\right)$ where $N_{D}$ is the number of the total epochs.

### 5.5. Effectiveness Evaluation

On four datasets, we examine the effectiveness of CLPNM-AD compared with that of six baseline methods. We use bold font to indicate the best $F_{1}$ score and underlined font to indicate the second-best $F_{1}$ score.

Table 3 shows the average $F_{1}$ scores and standard deviations of CLPNM-AD as well as the baseline methods. The $F_{1}$ score of CLPNM-AD surpasses all baseline methods on all datasets. Specifically, CLPNM-AD performs significantly better than the baseline methods on Thyroid and Micius, which achieves 11.5% and 11.0% improvement compared to the sub-optimal approaches DAGMM and LOF. For Satellite and Satimage datasets, CLPNM-AD still surpasses the next best method DAGMM and GOAD by 3.5% and 0.8%, respectively.

OC-SVM maps samples to one side of the hyperplane using a kernel function and does not take into account the semantic information shared by different samples. Simply mapping these samples to one side of the hyperplane tends to result in the overlapping of samples and thus makes it difficult to distinguish between normal and abnormal samples. Therefore, the results in Table 3 show that OC-SVM performs poorly among all methods.

LOF uses the distance between a sample and its surrounding points for anomaly detection, which takes into account the local similarity of the samples and to some extent the semantic similarity information of the samples. As a result, LOF outperforms OC-SVM, particularly on the Micius dataset, where OC-SVM performs suboptimally. However, because LOF is based on the original samples and lacks feature extraction capability, it cannot effectively model the data’s characteristics.

DAGMM uses the reconstruction error and intermediate layer vectors as feature vectors, followed by a Gaussian mixture model for anomaly detection, achieving good performance on most datasets because the mixture component of the Gaussian mixture model models the semantic category information of the samples. However, DAGMM performs poorly on the Micius dataset because the distinction between normal and abnormal samples was not very clear, and it cannot map different semantic category samples apart, resulting in poor performance.

DSVDD achieves anomaly detection by mapping samples into a hypersphere, but it lacks the ability to capture the semantic information of the samples and maps all samples into the same hypersphere, which results in samples with different semantic information overlapping in the feature space and does not facilitate the distinguishing of normal and abnormal samples. As can be seen from Table 3, DSVDD achieves poor performance on the Micius dataset, as does DAGMM.

GOAD augments the samples with a random affine transformation and then maps the augmented samples to the feature space for anomaly detection by predicting the transform used. The affine transform is used as the semantic class in this approach, and the augmented samples obtained using the same affine transform are clustered into one class in the feature space. Such an approach lacks sample semantic information modeling and achieves good results when the anomalous samples are of a single semantic category and the anomaly rate is low, e.g., as shown in Table 3, the model performs well on the Satimage dataset but performs poorly on the Satellite dataset where the anomalous samples contain more semantic categories and the anomaly rate is high.

In comparison to GOAD, NeuTraL AD employs a trainable multilayer neural network as the data augmentation function and a loss function similar to contrast loss to separate samples with different transformations for anomaly detection. The method suffers from the same limitation of a lack of sample semantic information modeling, and thus its performance is inadequate, as shown in Table 3.

Overall, CLPNM-AD outperforms all baselines on four datasets. The one-dimensional convolutional network in CLPNM-AD helps to capture the features of the samples, which can be adapted to anomaly detection tasks on different datasets. In addition, the consistency loss in CLPNM-AD helps the network to capture the semantic information of the samples, while the hard negative mixing strategy provides stronger guidance to help the contrast loss to separate and disperse the semantically different samples onto the hypersphere as much as possible, which allows the model to accurately profile the normal samples and thus make the normal and abnormal samples more distinguishable.

To quantitatively assess the effectiveness of CLPNM-AD, we statistically evaluate the results of 20 runs of CLPNM-AD with six baseline methods on four datasets using the Wilcoxon rank sum test. We test which of the null hypothesis H0 or the alternative hypothesis H1 holds for each pair of methods: H0: A ≈ B, H1: A > B, where A represents the $F_{1}$ score of CLPNM-AD on a specific dataset and B represents the $F_{1}$ score of the baseline method on the same dataset, A ≈ B means that the results of the two methods compared are not significantly different and A > B means that the results of A are better than those of B. For each test, we compute the *p*-value, and the hypothesis is tested at a significance level of $\alpha_{s}=0.01$.

As shown in Table 4, except for GOAD on Satimage, all Wilcoxon test results meet $p<\alpha_{s}$, allowing us to reject the null hypothesis H0 and accept the alternative hypothesis H1. This means that CLPNM-AD outperforms the baseline methods in all cases except for the Satimage dataset, where CLPNM-AD has nearly the same excellent results as GOAD.

In general, on all datasets, the CLPNM-AD method outperforms all baseline methods, and the advantage is statistically significant.

### 5.6. Robustness Evaluation

In satellite telemetry anomaly detection applications, the training data are frequently mixed with noisy data, or the anomalous samples are incorrectly labeled as normal during the training dataset construction, so we need to investigate the model’s sensitivity to contamination. In this experiment, we investigate how CLPNM-AD and all baselines respond to contaminated training data. We adopt the experiment setup in the literature [11] according to which, in each run, all of the anomaly data are combined with 50% of the randomly selected normal data to form the test set, and the remaining 50% of the normal data are mixed with c% of the anomaly data for model training. We examine the model’s average $F_{1}$ value when the contamination ratio is c%=[1%,2%,3%,4%,5%].

**Figure 4:**
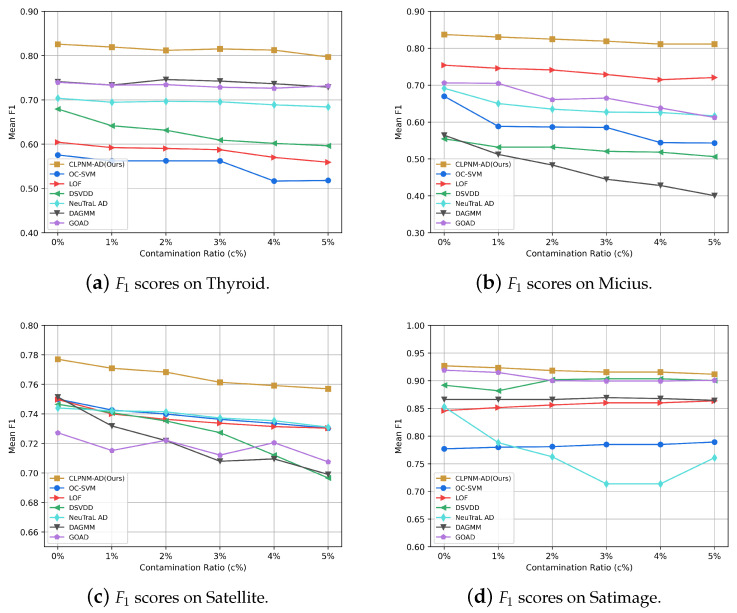
Anomaly detection results of CLPNM-AD and baseline methods on training datasets with contamination ratio *c*% = [0%, 1%, 2%, 3%, 4%, 5%].

Figure 4 illustrates the $F_{1}$ score of CLPNM-AD and all baselines after 20 runs. As expected, contaminated training data have a detrimental impact on detection accuracy in the majority of the situations. The mean $F_{1}$ score of CLPNM-AD declines somewhat across all datasets when the contamination ratio c% grows from 1% to 5%. When the contamination ratio is increased from 0 to 5%, the performance of CLPNM-AD decreased by 3.51%, 3.08%, 2.57% and 1.64% on the Thyroid, Micius, Satellite and Satimage datasets, respectively. Such a performance decline is somewhat tolerable. We also note that CLPNM-AD outperforms the baseline methods at different contamination ratios on all datasets.

CLPNM-AD has a certain tolerance for contaminated data, which may be because the consistency loss allocates the samples in a batch equally into each semantic category, preventing contaminated samples from being assigned to the same cluster. As a result, the contaminated samples have less impact on the probability distribution calculation of each semantic category, making the model somewhat robust to the anomalous samples mixed in the training set.

In general, CLPNM-AD is robust against contaminated data, maintaining good performance as the contamination ratio c% increases from 1% to 5% and always outperforming the performance of all baselines. In addition, to concentrate the model on the anomaly detection task and obtain greater anomaly detection accuracy, the model must be trained with a high-quality dataset, i.e., a clean dataset or a dataset with a low contamination ratio.

### 5.7. Ablation Experiment

In this experiment, we investigate the impact of various modules in the model. We repeat our experiment with/without a specific key module. These key modules are primarily vanilla InfoNCE loss $L_{infonce}$, clustering consistency objective loss $L_{consistency}$ and the negative samples mixing strategy, where $L_{infonce}$ and negative samples mixing strategy form $L_{pcl}$.

As shown in Table 5, our full model achieves the best performance on all datasets. When only $L_{consistency}$ is used, the method performs poorly because it makes no sense to emphasize consistency without the similarity information between samples as a semantic category guide. When only $L_{infonce}$ is used, the method performs better than when only $L_{consistency}$ is used because $L_{infonce}$ can explore the consistency of positive samples in the feature space while also pushing negative samples away, resulting in a more conducive semantic structure to detect anomalies. Combining $L_{consistency}$ and $L_{infonce}$ for model training outperforms $L_{consistency}$ and $L_{infonce}$ alone on most datasets. This is probably because $L_{consistency}$ can further exploit the semantic similarity information extracted by $L_{infonce}$ to make the clusters more separable and thus facilitate anomaly detection. Our full model achieves the best performance due to the ability of hard negative samples to contribute a larger magnitude gradient to update the network parameters, while also weakening the contribution of false negative samples to the gradient, preventing semantic similarity from being broken and thus facilitating the separation of normal and abnormal samples. In conclusion, these ablation experiments validate that each module in our model is useful and necessary.

### 5.8. Sensitivity Studies

In this section, we discuss the effect of the number of prototypes *K* and batch size *N* on the performance of CLPNM-AD on the Thyroid, Micius, Satellite and Satimage datasets.

**Figure 5:**
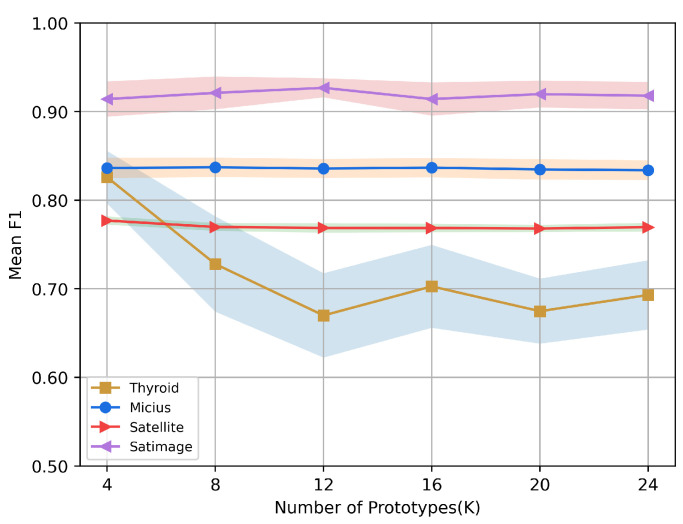
Sensitivity analysis for the number of prototypes.

Figure 5 depicts the performance of CLPNM-AD with varying numbers of prototypes K=[4,8,12,16,20,24]. It can be observed that CLPNM-AD is resistant to prototype numbers on Micius, Satellite and Satimage. In the case of Thyroid, the number of prototypes has an evident effect on CLPNM-AD, with $F_{1}$ decreasing as *K* increases from 4 to 24. We conjecture that the model degeneration on Thyroid is due to the fact that the dataset itself has specific semantic clusters, the number of which is close to four, and increasing the number of prototypes too much will break this semantic structure.

**Figure 6:**
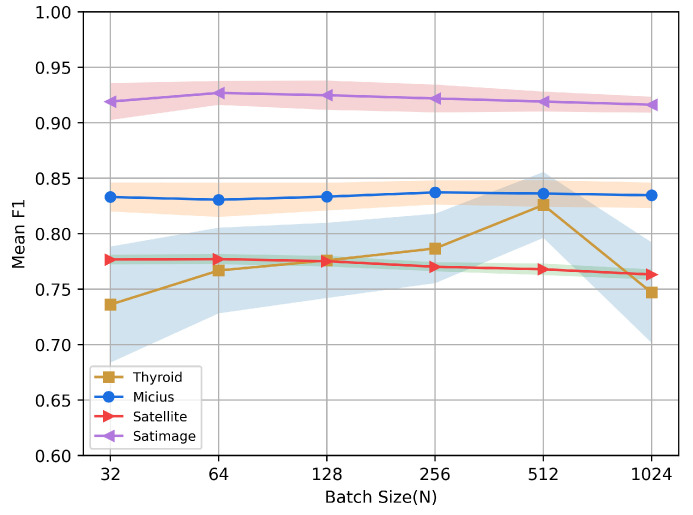
Sensitivity analysis for batch size.

Figure 6 illustrates the detection performance of CLPNM-AD with batch size N=[32,64,128,256,512,1024]. On Micius, Satellite and Satimage, we can see that CLPNM-AD is not sensitive to batch size, and the performance does not vary significantly when the batch size *N* is varied. On Thyroid, however, CLPNM-AD was more clearly affected by the batch size, with the method achieving the best performance at batch size N=512. This may be because too small batches contain too few samples and lack negative samples to guide network learning, while too large batches cause the network to converge more slowly and fail to fully converge for a given epoch number.

## 6. Conclusions

In this paper, we propose a correlation anomaly detection method for telemetry data, namely CLPNM-AD. The CLPNM-AD framework first employs an augmentation process with random feature corruption to obtain augmented samples. Following that, a prototype consistency strategy is used to capture the semantic category information of the samples. After that, prototype-based negative mixing contrastive loss is used to pull positive samples from the same prototype closer together and to push samples from different prototypes farther apart to obtain a profile that makes normal and anomaly distinguishable. Finally, anomaly decisions are made using a prototype-based anomaly score function. Extensive experiments are carried out to demonstrate superior performance to baselines on a variety of datasets, with up to 11.5% improvements on the standard $F_{1}$ score. The experiments also show CLPNM-AD’s robustness against noise, insensitivity to batch size and the number of prototypes and validate the need for each module in CLPNM-AD.

The method proposed in this paper combines contrastive learning and prototype learning to train an encoder to build a profile of normal telemetry data and then use that profile to distinguish between normal and anomalous samples, allowing for better anomaly detection. Our contribution is to use the distance between prototypes as a weight to synthesize hard negative samples and guide encoder learning, resulting in a more discriminative normal profile and a new framework for detecting correlation anomalies in telemetry parameters. In our real-world satellite operation tasks, the method complements existing limit checking and expert system approaches.

The results of the ablation experiments in Section 5.7 show that using the hard-negative samples synthesis strategy improves the algorithm’s performance on different datasets to varying degrees, and that using the learned semantic category information to guide the encoder’s learning is beneficial for learning normal profiles. It can also be seen that the degree of algorithm improvement varies across datasets, which may be related to the underlying semantic categories in the data, and thus exploring how to better design prototype learning algorithms may be an interesting direction for future research. Furthermore, the goal of anomaly detection is to create a profile that distinguishes between normal and anomalous samples, and in the future, adding stronger constraints to the normal profile (using synthetic anomalous samples or a small number of real anomalous samples) to learn a more discriminative normal profile should be considered. Simultaneously, practical applications in satellite operations necessitate the construction of anomaly detection models as simply and rapidly as possible, so future work should investigate how to automate the search for hyperparameters and the network structure of the encoder.

## Figures and Tables

**Table 1:** Statistical information of the datasets.

Dataset	Dimensions	Instances	Anomaly Ratio (ρ)
Thyroid	6	3772	0.025
Micius	19	11250	0.200
Satellite	36	6435	0.316
Satimage	36	5803	0.012

**Table 2:** Network structure of CLPNM-AD for each dataset.

	Operation	Units				Activation Function
		Thyroid	Micius	Satellite	Satimage	
Encoder	Linear	(6, 128)	(19, 128)	(36, 128)	(36, 128)	LeakyReLU(0.2)
	Reshape	(*, 16, 8)	(*, 16, 8)	(*, 16, 8)	(*, 16, 8)	
	Conv1d	(16, 32, 1)	(16, 32, 1)	(16, 32, 1)	(16, 32, 1)	
	BatchNorm1d	(32)	(32)	(32)	(32)	LeakyReLU(0.2)
	Conv1d	(32, 6, 1)	(32, 32, 1)	(32, 36, 1)	(32, 256, 1)	
	BatchNorm1d	(6)	(32)	(36)	(256)	LeakyReLU(0.2)
	Conv1d	(6, 6, 1)	(32, 32, 1)	(36, 36, 1)	(256, 256, 1)	
	BatchNorm1d	(6)	(32)	(36)	(256)	
	Flatten					LeakyReLU(0.2)
	Linear	(48, 6)	(256, 32)	(288, 36)	(2048, 256)	LeakyReLU(0.2)
Projection Head	Linear	(6, 6)	(32, 32)	(36, 36)	(256, 256)	
Prototypes (C)	Linear	(6, 4)	(32, 8)	(36, 4)	(256, 12)	

The symbol * in this table denotes a positive integer determined by the number of samples in the batch.

**Table 3:** Mean $F_{1}$ and σ of CLPNM-AD and all baseline methods(%). The highest $F_{1}$ scores are shown in bold, and the next highest $F_{1}$ scores are underlined.

Method	Dataset			
	Thyroid $F_{1}\pm \sigma$	Micius $F_{1}\pm \sigma$	Satellite $F_{1}\pm \sigma$	Satimage $F_{1}\pm \sigma$
OC-SVM [23]	57.6±4.5	67.0±0.7	75.0±0.4	77.7±3.1
LOF [25]	60.4±3.3	$\underline{75.4}\;\pm \;1.4$	75.0±0.7	84.6±2.6
DAGMM [11]	$\underline{74.1}\;\pm \;8.9$	56.4±7.2	$\underline{75.1}\;\pm \;3.6$	86.6±3.1
DSVDD [26]	67.9±6.0	55.5±0.7	74.7±0.4	89.2±2.3
GOAD [17]	74.0±3.7	70.6±6.0	72.7±1.8	$\underline{91.9}\;\pm \;2.2$
NeuTraL AD [18]	70.4±2.6	69.1±5.9	74.4±0.3	85.3±4.9
CLPNM-AD (Ours)	82.6±3.0	83.7±1.1	77.7±0.5	92.7±1.3

**Table 4:** *p*-values of Wilcoxon rank sum test for $F_{1}$.

Dataset	Method					
	OC-SVM	LOF	DAGMM	DSVDD	GOAD	NeuTraL AD
Thyroid	$3.26\times 10^{-8}$	$3.25\times 10^{-8}$	$1.69\times 10^{-6}$	$3.79\times 10^{-8}$	$3.71\times 10^{-7}$	$3.52\times 10^{-8}$
Micius	$3.39\times 10^{-8}$	$3.38\times 10^{-8}$	$3.39\times 10^{-8}$	$3.39\times 10^{-8}$	$4.57\times 10^{-8}$	$3.39\times 10^{-8}$
Satellite	$3.36\times 10^{-8}$	$3.37\times 10^{-8}$	$6.89\times 10^{-3}$	$3.36\times 10^{-8}$	$3.37\times 10^{-8}$	$3.34\times 10^{-8}$
Satimage	$2.60\times 10^{-8}$	$2.60\times 10^{-8}$	$3.52\times 10^{-8}$	$1.85\times 10^{-8}$	$1.33\times 10^{-1}$	$8.20\times 10^{-8}$

**Table 5:** Ablation study on Thyroid, Micius, Satellite and Satimage datasets.

$L_{\mathrm{consistency}}$		$L_{\mathrm{pcl}}$	Datasets			
	$L_{\mathrm{infonce}}$	Negatives Mixing	Thyroid	Micius	Satellite	Satimage
✔			63.3 ± 5.0	83.2 ± 1.1	74.8 ± 0.3	91.4 ± 1.0
	✔		75.4 ± 3.5	83.1 ± 1.3	75.5 ± 8.3	92.6 ± 1.9
✔	✔		80.3 ± 1.9	83.2 ± 1.2	75.7 ± 0.6	91.5 ± 2.5
✔	✔	✔	82.6 ± 3.0	83.7 ± 1.1	77.7 ± 0.5	92.7 ± 1.3

## Data Availability

Not applicable.
